# Tn*5*/*7*-*lux*: a versatile tool for the identification and capture of promoters in Gram-negative bacteria

**DOI:** 10.1186/s12866-015-0354-3

**Published:** 2015-02-04

**Authors:** Steven T Bruckbauer, Brian H Kvitko, RoxAnn R Karkhoff-Schweizer, Herbert P Schweizer

**Affiliations:** Department of Microbiology, Immunology and Pathology, and Rocky Mountain Regional Center of Excellence for Biodefense and Emerging Infectious Diseases Research, Colorado State University, Fort Collins, 80523 CO USA; Department of Molecular Genetics and Microbiology, College of Medicine, University of Florida, Emerging Pathogens Institute, PO Box 100266, Gainesville, 32610-0266 FL USA; Present Address: Microbiology Doctoral Training Program, University of Wisconsin-Madison, Madison, 53706 WI USA; Present Address: MSU-DOE Plant Research Laboratory, Michigan State University, East Lansing, 48824 MI USA

**Keywords:** Imaging, Luciferase, Bioluminescent bacteria, Host range, Mini-Tn*5/7*-*Lux* vectors, *Lux* fusion vectors, Gram-negative bacteria

## Abstract

**Background:**

The combination of imaging technologies and luciferase-based bioluminescent bacterial reporter strains provide a sensitive and simple non-invasive detection method (photonic bioimaging) for the study of diverse biological processes, as well as efficacy of therapeutic interventions, in live animal models of disease. The engineering of bioluminescent bacteria required for photonic bioimaging is frequently hampered by lack of promoters suitable for strong, yet stable luciferase gene expression.

**Results:**

We devised a novel method for identification of constitutive native promoters in Gram-negative bacteria. The method is based on a Tn*5*/*7* transposon that exploits the unique features of Tn*5* (random transposition) and Tn*7* (site-specific transposition). The transposons are designed such that Tn*5* transposition will allow insertion of a promoter-less bacterial *luxCDABE* operon downstream of a bacterial gene promoter. Cloning of DNA fragments from luminescent isolates results in a plasmid that replicates in *pir*^+^ hosts. Sequencing of the *lux*-chromosomal DNA junctions on the plasmid reveals transposon insertion sites within genes or operons. The plasmid is also a mini-Tn*7-lux* delivery vector that can be used to introduce the promoter-*lux* operon fusion into other derivatives of the bacterium of interest in an isogenic fashion. Alternatively, promoter-containing sequences can be PCR-amplified from plasmid or chromosomal DNA and cloned into a series of accompanying mini-Tn*7*-*lux* vectors. The mini-Tn*5/7*-*lux* and mini-Tn*7*-*lux* vectors are equipped with diverse selection markers and thus applicable in numerous Gram-negative bacteria. Various mini-Tn*5/7*-*lux* vectors were successfully tested for transposition and promoter identification by imaging in *Acinetobacter baumannii*, *Escherichia coli*, and *Burkholderia pseudomallei*. Strong promoters were captured for *lux* expression in *E. coli* and *A. baumannii*. Some mini-Tn*7*-*lux* vectors are also equipped with *attB* sites for swapping of the *lux* operon with other reporter genes using Gateway technology.

**Conclusions:**

Although mini-Tn*5*-*lux* and mini-Tn*7*-*lux* elements have previously been developed and used for bacterial promoter identification and chromosomal insertion of promoter-*lux* gene fusions, respectively, the newly developed mini-Tn*5/7-lux* and accompanying accessory plasmids streamline and accelerate the promoter discovery and bioluminescent strain engineering processes. Availability of vectors with diverse selection markers greatly extend the host-range of promoter probe and *lux* gene fusion vectors.

**Electronic supplementary material:**

The online version of this article (doi:10.1186/s12866-015-0354-3) contains supplementary material, which is available to authorized users.

## Background

The combination of recent advances in imaging technologies and development of luciferase-based bioluminescent reporter strains provide a sensitive and simple non-invasive detection method (biophotonic imaging) for the study of diverse biological processes, as well as efficacy of therapeutic interventions, in live animal models of human and animal disease [1-6]. *In vivo* bioluminescence can be employed to determine initial locations of infections and spatial migration of bioluminescently labeled pathogens over a period of several days to weeks. This technology has been applied to study chronic soft-tissue *Pseudomonas aeruginosa* and *Staphylococcus aureus* biofilm infections [7-10], *P. aeruginosa* and *Proteus mirabilis* urinary tract infections [11], as well as catheter-associated endovascular infections [12], and others [13-16]. Biophotonic imaging also allows assessments of the *in vivo* efficacy of antibiotic therapy in real time in living animals [9,11,13,17-20]. Some caveats of biophotonic imaging are: 1) luciferase-catalyzed reactions require energy (in the form of ATP and FMNH_2_), oxygen and a specific fatty acid substrate [21] and therefore allow the detection of only live, metabolically active cells. Because of the oxygen requirement of luciferases, bacterial cells expressing luciferase in strictly anaerobic environments such as the gut were in some instances found to be non-luminescent [1,2,16]. However, such instances are rare and bioluminescence can be detected in harvested organs exposed to oxygen [16]. Furthermore, other authors reported luciferase expression in anaerobic bacteria, e.g. *Bifidobacterium breve* grown *in vitro* and *in vivo* [22], and luciferase-tagged bacteria in anaerobic environments such as tumors [23]; 2) to ensure stable maintenance during the course of infections in animals, the bioluminescent reporter must be integrated into the chromosome of the respective bacteria. Replicating plasmids carrying the *lux* operon have been evaluated for bioimaging studies, but their use is limited because they only allow short-term (<48 h) infections to be accurately monitored *in vivo* in animals due to plasmid loss or dilution in the absence of antibiotic selection [24]. Chromosomal integration of plasmids via homologous recombination has been employed for construction of bioluminescent strains but the resulting strains are potentially unstable in the absence of antibiotic selection [25]. Initially, stable chromosomal integration was achieved by random transposition of a mini-Tn*5*-*luxCDABE* element [7,11] or another suitable transposon carrying the *lux* operon [13], followed by antibiotic resistance selection and screening for cells exhibiting strong expression of luciferase activity from a chromosomal promoter. Consequences of employing random transposition are: 1) need for investment of considerable efforts to determine transposon insertion sites and fitness of the mutant bacteria; 2) integrated transposons cannot easily be recovered or transferred between different mutant backgrounds for meaningful comparative analyses because most bacteria lack efficient chromosomal gene transfer procedures, except for those for which transducing phages are available or that are naturally transformable [13]; and 3) lack of a universal promoter for *lux* gene expression across either Gram-negative or Gram-positive bacteria necessitates development of new bioluminescent strains for each bacterial species to be studied with this technology.

In some bacteria the first two issues have been largely addressed and can be circumvented by use of site-specific insertion elements [25-30]. However, construction of bioluminescent reporter strains is still one of the limiting factors of biophotonic imaging. The major unmet need is lack of suitable promoters for luciferase expression in different bacteria. In Gram-positive bacteria development of synthetic promoters for luciferase gene expression have been successful in some cases [22,27]. However, previous attempts by our laboratory to engineer synthetic promoters based on, for example, the *Escherichia coli lac* operon-*trp* operon hybrid promoter *Ptac* [31] for use in non-enteric bacteria were largely unsuccessful mostly because of the instability of many of the synthetic promoters. We have successfully used the P1 integron promoter [32,33] for driving luciferase gene expression in *Burkholderia* species [29] indicating that this promoter may be useful for high-level constitutive gene expression in other non-enteric bacteria.

The purpose of this study was to create a simple to use, yet highly versatile series of plasmids for use in Gram-negative bacteria that facilitate promoter discovery and capture, as well as the creation of stable, bioluminescent strains of bacteria. To do this, we combined several features of transposons Tn*5* [34] and Tn*7* [35].

Tn*5* transposes randomly in bacteria. Minimal requirements for transposition are a transposase that can be provided *in trans*, mosaic ends (MEs) and an antibiotic resistance selection marker [36]. A mini-Tn*5* transposon contains the 19 bp MEs flanking the selection marker and is located on a delivery plasmid that contains the transposase gene *tnpA* outside of the mini-Tn*5* element [36]. Cargo cloned on the mini-Tn*5* can be randomly transposed into bacterial chromosomes. In contrast to Tn*5*, Tn*7* transposes site-specifically in Gram-negative bacteria, notably to chromosomal *att*Tn*7* sites in the presence of the site-specific transposition pathway composed of TnsABCD [35]. Most Gram-negative bacteria contain only a single *att*Tn*7* site associated with the essential *glmS* gene (encoding glucosamine-6-phosphate synthase) [37-41]. However, some contain multiple *glmS* genes and thus multiple *att*Tn*7* sites [37,42,43]. In one instance, *Proteus mirabilis*, one *glmS*- and one non-*glmS*-associated *att*Tn*7* site was documented [44]. Minimal requirements for Tn*7* transposition are a transposase that can be provided *in trans*, Tn*7* left and right ends (Tn*7*L and Tn*7*R) and an antibiotic resistance selection marker [35,37,45]. Cargo cloned on the mini-Tn*7* element can be site- and orientation-specifically transposed into bacterial chromosomes in the presence of a plasmid that transiently expresses the Tn*7* transposase subunits TnsABCD [37].

In this study, we constructed and tested mini-Tn*5/7*-*lux* elements with diverse selection markers that allow promoter identification by random Tn*5*-mediated transposition into the chromosomes of diverse target bacteria and screening for cells exhibiting strong expression of luciferase activity from a chromosomal promoter. The promoters can then be captured by self-ligation of chromosomal DNA fragments which creates a plasmid carrying a mini-Tn*7* element that serves as a template for promoter identification by DNA sequencing, or by PCR amplification of promoter-containing fragments. In some instances, e.g. in the presence of short chromosomal DNA inserts or when recombination-deficient recipient strains are available, the mini-Tn*7*-*lux* elements can be transposed into other bacteria without further modification. Alternatively, promoter-containing DNA fragments can be subcloned into a series of accompanying mini-Tn*7*-*lux* delivery vectors with diverse selection markers.

## Results

### Overview of the mini-Tn*5/7-lux* promoter identification, capture, and mini-Tn*7*-*lux* tagging procedures

**Figure 1:**
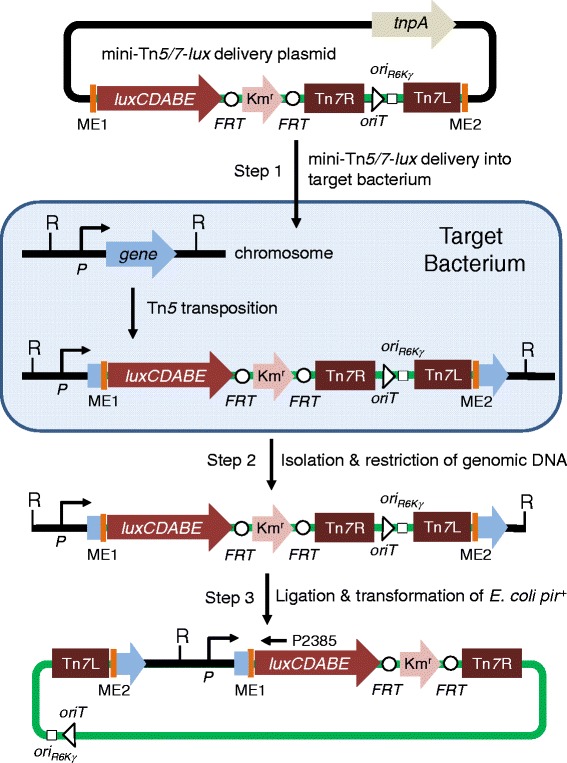
**Promoter identification and capture using mini-Tn** ***5/7-lux*** **elements.** Step 1 involves random Tn*5* transposition into the chromosome of the bacterial host of interest (target bacterium) after conjugal transfer of the mini-Tn*5/7*-*lux* delivery plasmid. This plasmid also contains the Tn*5* transposase encoding *tnpA* gene. TnpA acts on the Tn*5* mosaic ends (ME), which results in random mini-Tn*5/7*-*lux* transposition into the bacterial chromosome, including insertion in a gene where the *Photorhabdus luminescens luxCDABE* operon is transcribed from the gene’s promoter (*P*). The mini-Tn*7* element, i.e. sequences flanked by the Tn*7* left (Tn*7*L) and right (Tn*7*R) ends, located on the mini-Tn*5/7*-*lux* delivery plasmid does not transpose in this step because the delivery plasmid does not encode Tn*7* transposase. Mini-Tn*5/7*-*lux* chromosomal insertion is stable because the chromosomally-integrated elements do neither encode Tn*5* nor Tn*7* transposase. In step 2, chromosomal DNA of in this example kanamycin resistance (Km^r^) and light-emitting transformants is isolated and digested with a restriction enzyme (R) that does not cleave within the transposed element. In step 3, chromosomal DNA fragments are religated and a plasmid containing the R6K origin of replication (*ori*_R6K_) and origin of conjugal transfer (*oriT*) is recovered by transformation of a *pir* ^+^ *E. coli* host and selecting Km^r^ transformants. Sequencing of plasmid DNA with a *luxC*-specific primer (P2385) will reveal the transposon insertion site and putative promoter sequences. The Km^r^ selection marker contained on the chromosomally integrated mini-Tn*7*-*lux* elements is flanked by Flp recombinase target (*FRT*) sites for optional marker excision with *Saccharomyces cerevisiae* Flp recombinase.

**Figure 2:**
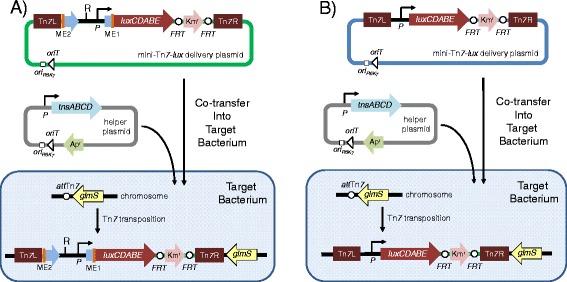
**Methods for site-specific insertion of promoter-** ***lux*** **fusions using mini-Tn** ***7-lux*** **elements.** Chromosomal integration of promoter-*lux* fusions can be achieved in two ways. **(A** **)** The plasmid recovered in step 3 of the promoter identification and capture procedure illustrated in Figure 1 is a functional mini-Tn*7* delivery plasmid which in some instances (e.g. when the plasmid contains short regions of promoter-containing chromosomal DNA or when RecA-deficient target strains are available) may be used to directly transpose site-specifically into the *glmS* gene-associated Tn*7* attachment site (*att*Tn*7*) in the chromosome of the bacterium under study to obtain luminescent derivatives. Site-specific mini-Tn*7*-*lux* insertion is achieved by co-transfer of the mini-Tn*7*-*lux* delivery plasmid and a helper plasmid that encodes the site-specific TnsABCD transposition pathway, which acts on the Tn*7* left (Tn*7*L) and right (Tn*7*R) ends. Both plasmids contain the origin of transfer (*oriT*) for conjugal transfer into the target bacterium and the conditional R6K origin of replication (*ori*_R6K_), which limits their replication to *E. coli* hosts expressing the plasmid R6K π protein. The mini-Tn*5* element (sequences flanked my the mosaic ends, ME) contained on the mini-Tn*7*-*lux* delivery plasmid does not transpose because the delivery plasmid does not encode Tn*5* transposase. **(B** **)** Alternatively, the promoter identified by sequencing the mini-Tn*5/7*-*lux-*chromosomal junction sequences located on the plasmid rescued in step 3 of the procedure illustrated in Figure 1 can be cloned into other mini-Tn*7*-*lux* elements. These are then transposed into the target bacterium for obtaining bioluminescent bacteria by site-specific mini-Tn*7*-*lux* transposition as described above. In both scenarios, **A** and **B**, the Km^r^ selection marker contained on the chromosomally integrated mini-Tn*7*-*lux* elements is flanked by Flp recombinase target (*FRT*) sites for optional marker excision with *Saccharomyces cerevisiae* Flp recombinase. The ampicillin resistance (Ap^r^) marker is used for selection and maintenance of the helper plasmid in *E. coli*.

The overall procedure involves promoter identification and capture (steps 1–3) (Figure 1) and, a unique aspect of the new procedure, methods for bacterial *lux* tagging by site-specific chromosomal insertion of promoter-*lux* fusions using mini-Tn*7*-*lux* elements (Figure 2). Promoter identification and capture comprises three steps. Step 1 involves Tn*5* transposition into the chromosome of the bacterial host of interest. This is achieved by conjugal transfer of the mini-Tn*5/7-lux* delivery plasmid, followed by selection of Km^r^ or other antibiotic resistance markers and screening for light-emitting exconjugants. Because the mini-Tn*5/7-lux* delivery plasmid is non-replicative in the host and Tn*5* transposase is only transiently transcribed from the plasmid backbone, the resulting bioluminescent bacteria have the mini-Tn*5/7-lux* element stably integrated such that the *lux* operon is transcribed from the promoter(s) of the target gene containing the mini-Tn*5/7*-*lux* insertion. This gene can either be a single transcriptional unit or part of an operon. To identify the mini-Tn*5/7-lux* insertion site, genomic DNA is digested with an enzyme that does not cleave within the transposed element (step 2). Since mini-Tn*5/7-lux* contains an *ori*_R6K_ and an antibiotic resistance selection marker, religation of the DNA fragment containing the transposon results in a plasmid which can be recovered by transformation of a *pir*^+^*E. coli* host and selecting antibiotic resistant transformants (step 3). Sequencing of plasmid DNA with a *luxC*-specific primer (P2385) will reveal the transposon insertion site. Tn5 transposition is not affected by the target bacterium’s recombination status because the mini-Tn*5/7*-*lux* plasmid does not carry any chromosomal DNA.

The captured promoter transcribing the *lux* gene operon can be used to derive bioluminescent bacteria by tagging with mini-Tn*7*-*lux* elements in two ways. The choice of method is in part affected by the target bacterium’s recombination status and the size of promoter-containing DNA fragment. First, the mini-Tn5/7-*lux* delivery plasmid used for promoter identification and capture is designed such that the plasmid recovered in step 3 of the promoter identification and capture procedure illustrated in Figure 1 is a functional mini-Tn*7* delivery plasmid which in some instances (e.g. when the plasmid contains short regions of promoter-containing chromosomal DNA or when RecA-deficient target strains are available) may be used to directly transpose site-specifically into the *glmS* gene-associated Tn*7* attachment site (*att*Tn*7*) in the chromosome of the bacterium under study to obtain luminescent derivatives. For site-specific transposition, mini-Tn*7* elements require the Tn*7* transposase complex, which is encoded by a helper plasmid containing the *tnsABCD* genes specifying the site-specific Tn*7* transposition pathway. Second, the plasmid recovered in step 3 of the promoter identification and capture procedure illustrated in Figure 1 may be used as a source for promoter-containing DNA fragments that can be PCR amplified, cloned into other mini-Tn*7*-*lux* elements, and be employed for obtaining bioluminescent bacteria after site-specific mini-Tn*7*-*lux* transposition as described above. This procedure is advised when the plasmid obtained in the promoter recovery step contains larger (several kb) regions of promoter-containing chromosomal DNA or when RecA-deficient target strains are not available. In these instances, chromosomal integration via homologous recombination is favored over site-specific mini-Tn*7* integration. Examples for both mini-Tn*7*-*lux* tagging scenarios are presented below. Direct tagging with a mini-Tn*7*-*lux* element containing the captured promoter transcribing the *lux* operon is illustrated in a *recA E. coli* strain. A wild-type *Acinetobacter baumannii* strain is presented as an example for a bacterium tagged with a mini-Tn*7*-*lux* element where *lux* operon transcription is driven by a promoter which was identified during using mini-Tn*5/7*-*lux* mediated identification and capture, and then subcloned in a mini-Tn*7* element harboring a promoter-less *lux* operon.

It should be reiterated at this point that, as noted above, most Gram-negative bacteria contain only one chromosomal *glmS*-associated *att*Tn*7* site [37-41] with the exception of *Proteus mirabilis* [44]. In contrast, the majority of *Burkholderia* species examined to date contain multiple *glmS* genes and thus multiple *att*Tn*7* sites, ranging from two sites in *B. thailandensis* [37] and *B. mallei* [42] to three sites in *B. pseudomallei* [43]. Although insertions in these bacteria can occur at all sites, most insertions are usually at one, preferred *att*Tn*7* site. In *B. mallei*, analysis of 24 randomly selected insertions showed that 96% of the insertions were at the *glmS1*-associated *att*Tn*7* site. By contrast, only 8% of the insertions were at the *glmS2*-associated *att*Tn*7* site. Only 4% of the transformants had insertions at both *glmS1* and *glmS2* [42]. In *B. pseudomallei*, >65% of observed insertions occur at the *glmS2*-associated *att*Tn*7* site, but there is no obvious preference for either the *glmS1*- or *glmS3*-associated *att*Tn*7* sites. While double insertions in two separate *att*Tn*7* sites are fairly common (10 to 20% with some strains), triple insertions are rarely observed [43]. Presence of multiple *att*Tn*7* sites is not an impediment because sites of insertions can be readily differentiated by multiplex PCR. An example for insertion site analysis in *B. pseudomallei* is illustrated in Additional file 1: Figure S3.

### Tn*5*/*7-lux*-based promoter capture in *E. coli* DH5α

**Figure 3:**
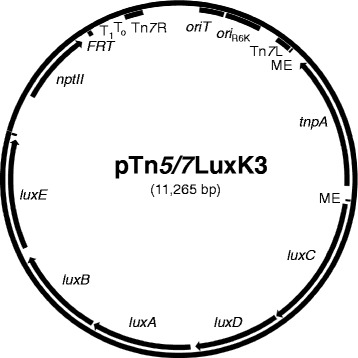
**Map of mini-Tn** ***5/7*** **-** ***lux*** **delivery plasmid pTn** ***5/7*** **LuxK3.** The mini-Tn*5/7*-*lux* element flanked by the Tn*5* mosaic ends (ME) carries a promoter-less *P. luminescens luxCDABE* operon, the kanamycin resistance encoding *nptII* gene, the Tn*7* left (Tn*7*L) and right (Tn*7*R) ends, the R6K origin of replication (*ori*_R6K_), and an origin of conjugative transfer (*oriT*). The Tn*5* transposase-encoding *tnpA* gene is located outside of the transposable element. Other abbreviations: *FRT*, single Flp recombinase target site; T_0_T_1_, transcriptional terminators T_0_ and T_1_ from bacteriophage λ and *E. coli rrnB* operon, respectively.

**Figure 4:**
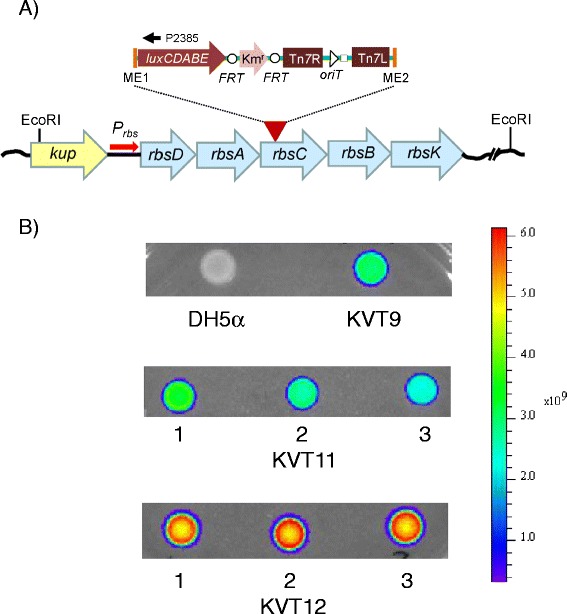
**Mini-Tn** ***5/7*** **-** ***lux*** **aided promoter identification and capture in** ***E. coli*** **. A)** The promoter-less mini-Tn*5*/*7*-*lux* element (indicated by the red arrowhead and expanded inset above it) from pTn*5/7*LuxK3 was transposed into the DH5α chromosome. Chromosomal DNA from a Km^r^ and luminescent exconjugant (KVT9; panel B) was isolated, digested with *Eco*RI, religated and transformed into CC118(λ*pir* ^+^). The *lux* operon-chromosomal DNA junction on the plasmid was sequenced with the *luxC*-specific primer P2385. The mini-Tn*5/7*-*lux* transposon was inserted in *rbsC*, the third gene of the *rbsDACBK* operon required for the transport and initial metabolic steps of ribose. The *kup* gene located upstream of the *rbsDACBK* operon encodes a potassium transporter. **B)** The recovered plasmid was used to transpose the mini-Tn*7*-*P*_*rbs*_-*lux* element residing on it to the *att*Tn*7* site on the DH5α chromosome resulting in strain KVT11. In a parallel effort, The *rbs* operon promoter (*P*_*rbs*_) located in the 167 bp *kup*-*rbsD* intergenic region was PCR-amplified and cloned on a 173 bp *Stu*I-*Dra*III fragment into pTn*5/7*LuxK4 where it replaced the *tnpA* gene and flanking MEs to drive transcription of the *lux* operon. The resulting mini-Tn*7*-*P*_*rbs*_-*lux* element was transposed into the *att*Tn*7* site on the DH5α chromosome resulting in strain KVT12. Ten μl samples of an overnight culture of the indicated strains were spotted on an LB plate, grown overnight at 37°C and light emission was measured using a Xenogen IVIS imager.

To assess the feasibility of the mini-Tn*5/7*-lux procedure, we constructed the first member of the mini-Tn*5/7*-*lux* family, pTn5/7-LuxK3 (Figure 3). Transposition of pTn*5*/7-LuxK3 into DH5α after conjugal transfer and selection of Km^r^ exconjugants produced approximately 1% brightly luminescent clones amongst the colonies that were examined for luminescence. A bright isolate (KVT9) was chosen for further study. Genomic DNA was isolated, digested with *Eco*RI and self-ligated fragments were used to transform *E. coli pir*^+^ strain CC118(λ*pir*^+^) [46]. Km^r^ transformants were screened for light emission and a plasmid containing the captured promoter region, pTn7_DH51_LuxK3, was isolated. The mini-Tn*5/7-lux* insertion site was determined to be located in the *rbsC* gene, the third gene of the *rbsDACBK* operon required for the transport and initial steps of ribose metabolism [47] (Figure 4A). The mini-Tn*7-P*_*rbs*_-*lux* element contained on pTn7_DH51_LuxK3 was transposed into the DH5α chromosome and mini-Tn*7* transposition was confirmed by PCR. Since DH5α is a *recA* mutant strain all exconjugants examined contained the mini-Tn*7-P*_*rbs*_-*lux* element inserted at the *att*Tn*7* site instead of recombination-mediated insertion at the *rbsDACBK* locus. Imaging showed that the resulting strain (KVT11) emitted similar levels of light when compared to the original mini-Tn*5/7-lux* transposition clone KVT9 (Figure 4B). To ascertain that the *rbsDACBK* operon promoter (*P*_*rbs*_) located in the 167 bp *kup*-*rbsD* intergenic region was responsible for *lux* operon transcription in strain KVT9 and KVT11, the predicted *rbs* promoter region was PCR-amplified on a 182-bp fragment and directionally cloned upstream of the *lux* operon contained on pTn*5/7*LuxK4 resulting in pTn*7P*_*rbs*_LuxK4. Transposition of the resulting mini-Tn*7-P*_*rbs*_-*lux* element into DH5α resulted in strain KVT12 which was more bioluminescent than KVT9 and KVT11 presumably because the *rbs* promoter was placed closer to the *lux* operon on the mini-Tn*7-P*_*rbs*_-*lux* element inserted in KVT12 (Figure 4B).

### Construction of next generation *lux* vectors

**Figure 5:**
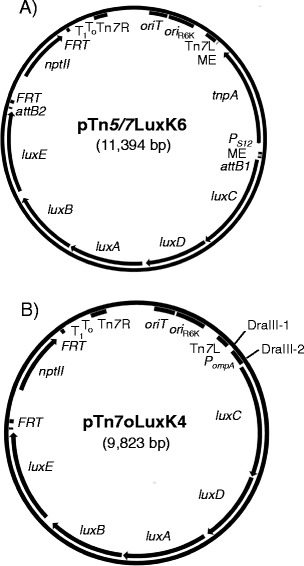
**Maps of next generation mini-Tn** ***5/7*** **-** ***lux*** **and mini-Tn** ***7*** **-** ***lux*** **delivery vectors. A)** pTn*5/7*LuxK6**.** As in pTn*5/7*LuxK3 (Figure 2) the mini-Tn*5/7*-*lux* element is flanked by the Tn*5* mosaic ends (ME) and carries a promoter-less *P. luminescens luxCDABE* operon, in this example the kanamycin resistance encoding *nptII* gene, the Tn*7* left (Tn*7*L) and right (Tn*7*R) ends, the R6K origin of replication (*ori*_R6K_), and an origin of conjugative transfer (*oriT*). The new generation of vectors is further functionalized by 1) transcription of the Tn*5* transposase-encoding *tnpA* gene located outside of the transposable element by the constitutive S12 gene promoter (*P*_*S12*_) from *Burkholderia thailandensis*; 2) flanking of the antibiotic resistance marker by functional Flp recombinase target (*FRT*) sites; and 3) flanking of the *lux* operon by *attB* sites for Gateway recombineering. The pTn*5/7*LuxG6, pTn*5/7*LuxT6 and pTn*5/7*LuxTc6 contain gentamicin, trimethoprim and tetracycline resistance markers, respectively. Other abbreviations are defined in the Figure 2 legend. **B)** pTn*7*oLuxK4. This vector contains many features of the pTn*5/7*Lux series but does not contain the Tn*5* transposase gene or mosaic ends. Its unique features include two *Dra*III sites that because of the 3-nucleotide ambiguity (indicated by bold letters) in the *Dra*III recognition sites (*Dra*III-1 5′-CACTATGTG and *Dra*III-2 5′- CACCGCGTG) can be used for directional cloning of promoter-containing DNA fragments for transcription of the *lux* operon genes. In the illustrated example the *lux* operon is transcribed from the *B. pseudomallei ompA* promoter (*P*_*ompA*_). This is also indicated by the “o” in the plasmid name. The pTn*7*oLuxG4 and pTn*7*oLuxT4 contain *P*_*ompA*_ and the gentamicin (*aacC1*) and trimethoprim (*dhfRII*) resistance markers, respectively. Other abbreviations are as in **A)**.

After successful testing in *E. coli*, we sought to expand the versatility of the mini-Tn*5/7*-*lux* system for use in a broad-range of bacteria and by inclusion of other desirable properties such as incorporation of *attB1* and *attB2* sites flanking the *lux* operon to facilitate its exchange for other reporter genes such as *gfp* via Gateway technology [48]. This resulted in a family of versatile plasmids with diverse selection markers (gentamicin, kanamycin, tetracycline and trimethoprim) most of which can be excised *in vivo* using Flp recombinase, a *lux* operon with or without exchangeable promoters and the Tn*5* transposase *tnpA* gene with flanking MEs either transcribed from its own promoter or the constitutive S12 gene promoter from *B. thailandensis* (Table 1). A graphical representation of the genealogy of the various plasmids is presented in Additional file 1: Figure S1 and detailed maps of two representative plasmids, pTn*7*oLuxK4 and pTn*5/7*LuxK6, are shown in Figure 5.

**Table 1:** **Plasmids**

**Plasmid**	**GenBank accession no.**	**Pertinent features** ^**a,b**^	**Source**
pTn*7*xLuxG0	KF532964	Gm^r^; *luxCDEBA* operon transcribed from *P*_*PA4974*_ contained on DraIII fragment	This Study
pTn*7*oLuxG0	KF532961	Gm^r^; DraIII fragment containing *P*_*PA4974*_ replaced with DraIII fragment containing *P*_*ompA*_^c^	This Study
pTn*7*xLuxG3	KF532965	Km^r^; pTn*7*xLuxG0 with BamHI and PstI sites deleted by partial digestion	This Study
pTn*7*xLuxK3^d^	KC332283	Km^r^; pTn*7*xLuxG3 with Gm^r^ encoding *aacC1* gene replaced with Km^r^ encoding *nptII* gene from pFKM4	This Study
pTn*7*oLuxK3^d^	KC332281	Km^r^; pTn*7*xLuxK3 with DraIII fragment containing *P*_*PA4974*_ replaced with DraIII fragment containing *B. pseudomallei P*_*ompA*_	This Study
pTn*7*tLuxK3^d^	KC332282	Km^r^; pTn*7*xLuxK3 with DraIII fragment containing *P*_*PA4974*_ replaced with DraIII fragment containing *B. pseudomallei P*_*tolC*_	This Study
pTn*5/7*LuxK3^d^	KC332286	Km^r^; pTn*7*xLuxK3 with StuI-DraIII fragment containing *P*_*PA4974*_ replaced with StuI-DraIII fragment containing Tn*5* transposase gene *tnpA* and flanking mosaic ends	This Study
pTn*7*oLuxG4	KF532962	Gm^r^; pTn*7*oLuxG0 with BamHI multiple cloning site fragment deleted	This Study
pTn*7*oLuxK4	KC332284	Km^r^; pTn*7*oLuxG4 with Gm^r^ encoding *aacC1* gene replaced with Km^r^ encoding *nptII* gene from pFKM4	This Study
pTn*7*oLuxT4	KF532963	Tp^r^; pTn*7*oLuxG4 with Gm^r^ encoding *aacC1* gene replaced with Tp^r^ encoding *dhfRII* gene from pFTP2	This Study
pTn*5/7*LuxG4	KF813061	Gm^r^; pTn*7*oLuxG4 with StuI-DraIII fragment containing *P*_*ompA*_ replaced with StuI-DraIII fragment containing Tn*5* transposase gene *tnpA* and flanking mosaic ends	This Study
pTn*5/7*LuxK4	KF532957	Km^r^; pTn*7*oLuxK4 with StuI-DraIII fragment containing *P*_*ompA*_ replaced with StuI-DraIII fragment containing Tn*5* transposase gene *tnpA* and flanking mosaic ends	This Study
pTn*5/7*LuxT4	KF813062	Tp^r^; pTn*7*oLuxT4 with with StuI-DraIII fragment containing *P*_*ompA*_ replaced with StuI-DraIII fragment containing Tn*5* transposase gene *tnpA* and flanking mosaic ends	This Study
pTn*5/7*LuxK5	KF532958	Km^r^; pTn*5/7*LuxK4 with *tnpA* gene transcribed from *P*_*S12*_	This Study
pTn*5/7*LuxT5	KF532959	Tp^r^; pTn*5/7*LuxT4 with *tnpA* gene transcribed from *P*_*S12*_	This Study
pTn*5/7*LuxT6	KC332285	Tp^r^; pTn*5/7*LuxT5 with *attB1* inserted at DraIII site	This Study
pTn*5/7*LuxG6	KC332289	Gm^r^; pTn*5/7*LuxT6 with Tp^r^ encoding *dhfRII* gene replaced with Gm^r^ encoding *aacC1* gene from pFGM1	This Study
pTn*5/7*LuxK6	KC332287	Km^r^; pTn*5/7*LuxT6 with Tp^r^ encoding *dhfRII* gene replaced with Km^r^ encoding *nptII* gene from pFKM4	This Study
pTn*5/7*LuxTc6	KC332288	Tc^r^; pTn*5/7*LuxT6 with Tp^r^ encoding *dhfRII* gene replaced with Tc^r^ encoding *tetA* gene from pFTC2	This Study
pTn*7P*_*DH51*_-*lux*	N/A	Km^r^; captured DH5α *rbsC*::Tn*5*/*7*LuxK3 insertion on mini-Tn*7* delivery plasmid obtained by religation of chromosomal Acc65I fragment	This Study
pTn*7P*_*rbs*_LuxK4	N/A	Km^r^; *E. coli rbs* promoter (*P*_*rbs*_) cloned into pTn7LuxK4	This Study
pTn*7P*_*H1A*_LuxK5	N/A	Km^r^; *A. baumannii* A1S_0945 gene promoter (*P*_*H1A*_) cloned into pTn*7*LuxK5	This Study

^a^Abbreviations: Ap, ampicillin; *FRT*, Flp recombinase target; Gm, gentamicin; Km, kanamycin; r, resistance/resistant; Tc, tetracycline; Tp, trimethoprim.

^b^See supplemental methods for details of plasmid constructions.

^c^Promoters used are: *P*_*PA4974*_, *P. aeruginosa* gene PA4974 promoter; *P*_*ompA*_, *B. pseudomallei ompA* promoter; *P*_*tolC*_, *B. pseudomallei tolC* promoter; *P*_*S12*_, *B. thailandensis* ribosomal S12 gene promoter.

^d^Plasmids are missing a *FRT* site flanking the *nptII* gene.

### Tn*5*/*7*-*lux* based promoter capture in *A. baumannii*

**Figure 6:**
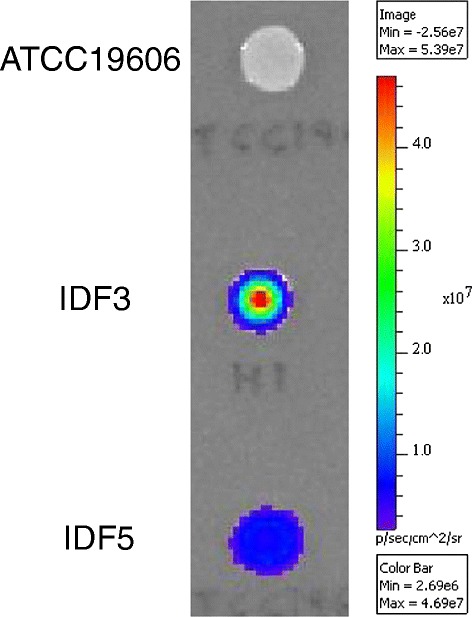
**Mini-Tn** ***5/7*** **-** ***lux*** **aided promoter identification and capture in** ***A. baumannii*** **.** The promoter-less mini-Tn*5*/*7*-*lux* element from pTn*5/7*LuxK5 was transposed into the *A. baumannii* strain ATCC19606 chromosome. Chromosomal DNA from a Km^r^ and luminescent exconjugant (IFD3) was isolated, digested with *Acc*65I, religated and transformed into *E. coli pir-116* ^+^ strain MaH1. DNA from a Km^r^ transformants was sequenced with the *luxC*-specific primer P2385. The mini-Tn*5/7*-*lux* transposon was inserted in a gene annotated as A1S_0947 in strain ATCC17978 and the upstream A1S_0944-A1S_0945 intergenic region containing several possible promoters was PCR-amplified and cloned on a 329-bp *Stu*I-*Dra*III fragment into pTn*5/7*LuxK5. The mini-Tn*7*-*P*_*H1A*_-*lux* element was transposed into the *att*Tn*7* site on the ATCC19606 chromosome resulting in strain IFD5. Ten μl samples of an overnight culture of the indicated strains were spotted on an LB plate, grown overnight at 37°C and light emission was measured using a Xenogen IVIS imager.

To demonstrate promoter identification and recovery in a bacterium other than *E. coli*, pTn*5/7*LuxK5 was conjugated into *A. baumannii* strain ATCC19606. From the pool of recovered Km^r^ exconjugants, about 1% exhibited strong light emission. Four luminescent strains (IFD1-4) were retained for further studies. To identify mini-Tn*5/7*-*lux* insertion sites in these strains, chromosomal DNA was isolated and digested with *Acc*65I, *Eco*RI and *Not*I. These enzymes were empirically chosen because they cleave chromosomal DNA with various frequencies in different bacteria based on G + C content and their capacity for heat inactivation. For instance, in *A. baumannii* the chromosomal DNA cleavage frequency decreased from *Acc*65I to *Eco*RI to *Not*I. Plasmids harboring the mini-Tn*5/7*-*lux* elements were obtained after self-ligation and transformation of *E. coli pir-116*^+^ strain MaH1 (Table 2). The transposon-chromosomal junction sequences were determined by sequencing and aligning the sequences thus obtained to the chromosomal sequence (EMBL accession no. CP000521) of *A. baumannii* strain ATCC17978. These analyses revealed single mini-Tn*5/7*-*lux* insertions in isolate IFD1 (gene A1S_2680), IFD2 (gene A1S_2773) and IFD4 (gene A1S_2621) and two insertions in strain IFD3 (genes A1S_0947 and A1S_2736). In an attempt to insert the respective mini-Tn*7*-*lux* elements located on the respective delivery plasmids into the *A. baumannii* chromosomal Tn*7* attachment site they were conjugally transferred from *E. coli* RHO3 into strain ATCC19606 together with the helper plasmid pTNS3 [43]. Although luminescent exconjugants were observed, PCR screening of 80 colonies revealed no mini-Tn*7*-*lux* insertion at the *att*Tn*7* site but rather only strains in which the mini-Tn*7*-*lux* elements had integrated at the respective chromosomal loci via RecA-mediated homologous recombination. To minimize homologous recombination events, we identified potential promoter regions upstream of the previously identified mini-Tn*5/7*-*lux* insertion sites. These analyses revealed the presence of multiple potential promoters in the A1S_0944–A1S_0945 intergenic region that is located upstream of the mini-Tn*5/7*-*lux* insertion site in A1S_047 encoding a putative vanillate *O*-demethylase oxygenase subunit. The region containing the putative gene 0945 promoter region (*P*_*H1A*_) was PCR-amplified and directionally cloned into pTn*5/7*LuxK5 to create pTn*7P*_*H1A*_LuxK5. Km^r^*E. coli* MaH1 transformants containing the desired promoter insertion were identified by screening for increased luminescence as the pTn*5/7*LuxK5 vector with a promoter-less *lux* operon confers negligible luminescence. The mini-Tn*7*-*P*_*H1A*_-*lux* element was then transposed into the ATCC19606 chromosome. The resulting strain IFD5 containing the mini-Tn*7-P*_*H1A*_-*lux* transposon integrated at the chromosomal *att*Tn*7* site emitted slightly less light than the original strain IFD3, which contained two separate mini-Tn*5/7-lux* insertions (Figure 6).

**Table 2:** **Bacterial strains**

**Strain**	**Genotype or relevant features**	**Source**
*E. coli*		
DH5α	F^−^φ80 *lacZ*ΔM15 Δ(*lacZYA-argF*)U169 *deoR recA1 endA1 hsdR17*(r_K_^−^m_K_^+^) *phoA glnV44*	[49]
MaH1	F^−^φ80 *lacZ*ΔM15 Δ(*lacZYA-argF*)U169 *deoR recA1 endA1 hsdR17*(r_K_^−^m_K_^+^) *phoA glnV44 att*Tn*7*::*pir116*+	[50]
RHO3	*thi-1 thr-1 leuB26 tonA21 lacY1 supE44 recA* integrated RP4-2 Tc^r^::Mu (λ*pir* ^*+*^)Δ*asd*::*FRT* Δ*aphA*::*FRT*	[51]
RHO5	*att*Tn*7::pir116* ^*+*^ *thi-1 thr-1 leuB26 tonA21 lacY1 supE44 recA* integrated RP4-2 Tc^r^::Mu (λ*pir* ^+^)Δ*asd*::*FRT* Δ*aphA*::*FRT*	[50]
KVT9	DH5α *rbsC*::mini-Tn*5*/*7*LuxK3	This Study
KVT11	DH5α *att*Tn7::mini-Tn*7*-*P*_*DH51*_-*lux*	This Study
KVT12	DH5α *att*Tn7::mini-Tn7-P_*rbs*_-*lux*	This Study
*B. pseudomallei*		
Bp82	1026b Δ*purM*	[52]
Bp82.27	Bp82 Δ(*amrRAB-oprA*)	Laboratory collection
Bp82.68	Bp82.27 *att*Tn*7*::mini-Tn*7-P*_*ompA*_ *-lux* (*glmS1*)	This Study
Bp82.69	Bp82.27 *att*Tn*7*::mini-Tn*7-P*_*tolC*_ *-lux* (*glmS1*)	This Study
Bp82.70	Bp82.27*att*Tn*7*::mini-Tn*7-P*_*PA4974*_-*lux* (*glmS2 + glmS3*)	This Study
Bp82.77	Bp82.27*att*Tn*7*::mini-Tn*7-P*_*ompA*_ *-lux* (*glmS1* + *glmS2*)	This Study
Bp82.78	Bp82.27 *att*Tn*7*::mini-Tn*7-P*_*ompA*_ *-lux* (*glmS1*)	This Study
Bp82.79	Bp82.27 *att*Tn*7*::mini-Tn*7-P*_*ompA*_ *-lux* (*glmS2 + glmS3*)	This Study
Bp82.80	Bp82.27 *att*Tn*7*::mini-Tn*7-P*_*ompA*_ *-lux* (*glmS2*)	This Study
Bp82.81	Bp82.27 *att*Tn*7*::mini-Tn*7-P*_*ompA*_ *-lux* (*glmS3*)	This Study
*A. baumannii*		
ATCC19606	Prototroph	American Type Culture Collection
IFD1	ATCC19606 A1S_2680::mini-Tn*5*/*7*LuxK5	This Study
IFD2	ATCC19606 A1S_2773::mini-Tn*5*/*7*LuxK5	This Study
IFD3	ATCC19606 A1S_0947::mini-Tn*5*/*7*LuxK5 and A1S_2736::Tn*5*/*7*LuxK5	This Study
IFD4	ATCC19606 A1S_2621::mini-Tn*5*/*7*LuxK5	This Study
IFD5	ATCC19606 *att*Tn*7*::mini-Tn*7*-*P*_*H1A*_-LuxK5	This Study

### Luminescence from mini-Tn*7*-*lux* elements in bacteria with multiple *att*Tn*7* sites is insertion site dependent

Noting that bacteria which contain multiple mini-Tn*7*-*lux* insertions due to the presence of multiple Tn*7* insertion (*att*Tn*7*) sites exhibit differential luminescence we decided to examine light emission from bacteria which naturally contain more than one mini-Tn*7* insertion site and in which luminescence is thus either insertion site-dependent or due to multiple insertions.

**Figure 7:**
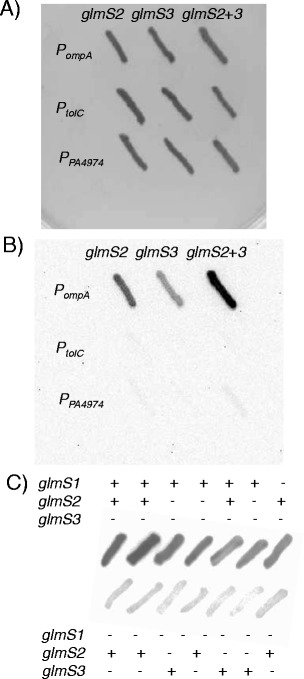
**Promoter strength and insertion site location determine bioluminescence signal strength in a bacterium with multiple Tn** ***7*** **insertion sites.** Mini-Tn*7* elements in which *lux* operon expression is directed from the indicated promoters were transposed into the genome of *B. pseudomallei* strain Bp82.27 and insertion at either *glmS1-*, *glmS2-* or *glmS3*-associated *att*Tn*7* sites was determined by PCR. Colonies were patched on LB agar plates with 35 μg/ml kanamycin and incubated at 37°C. Patches were imaged using a Bio-Rad Universal Hood II ChemiDocXRS using high sensitivity chemiluminescence settings. **A)** and **B)** Bp82.27 with mini-Tn*7*-*lux* insertions derived from transposition from pTn*7*oLuxK3, pTn*7*tLuxK3 and pTn*7*xLuxK3. The promoters directing *lux* operon expression in these constructs are *B. pseudomallei P*_*ompA*_, *B. pseudomallei P*_*tolC*_ and *P. aeruginosa P*_*PA4974*_, respectively. Patches were grown overnight at 37°C and either imaged using white light and a 0.007 s exposure time (A) or imaged for luminescence using a 30 s exposure time **(B)**. **C)** Insertion site dependence of bioluminescence intensity. The mini-Tn*7*-*P*_*ompA*_ *-lux* element from pTn*7*oLuxK4 was transposed into the Bp82.27 genome. Insertion sites were determined by PCR. Patches were grown overnight at 37°C and imaged for luminescence using a 30 s exposure time.

During isolation of *B. pseudomallei* strains containing mini-Tn*7*-*lux* elements we noticed that the resulting strains emitted various amount of light and this seemed to be promoter and insertion site dependent. In this study we therefore compared various promoters, including *P. aeruginosa P*_*PA4974*_ [7], *B. pseudomallei P*_*ompA*_ [28] and *P*_*tolC*_ [25] cloned upstream of the *lux* operon residing on pTn*7*oLuxK4 and inserted at diverse *att*Tn*7* sites in the genome of *B. pseudomallei* strain Bp82.27, an aminoglycoside susceptible Δ(*amrAB-oprA*) derivative of the select agent excluded strain Bp82 [52]. Kanamycin resistant transformants were patched onto Km-containing LB plates and light production was compared. In this system, light production from a *lux* operon transcribed by *P*_*ompA*_ was consistently strongest followed by *P*_*PA4974*_ and *P*_*tolC*_ (Figure 7A and B). Mini-Tn*7*-*lux* insertion into the *glmS*1-associated *att*Tn*7* site consistently conferred the greatest luminescence while strains carrying single mini-Tn*7*-*P*_*ompA*_-*lux* insertions into the *glmS*2-associated or *glmS*3-associated *att*Tn*7* sites produced consistently less luminescence (Figure 7C). A strain carrying simultaneous insertions of mini-Tn*7*-*P*_*ompA*_-*lux* in all three *B. pseudomallei att*Tn*7* sites was not observed.

## Discussion

The mini-Tn*5/7*-*lux* vectors were employed to successfully identify, capture and clone promoters capable of producing significant amounts of light in *E. coli* and *A. baumannii* under laboratory conditions. While in this study efforts were focused on vector construction and evaluation *in vitro*, future efforts must include studies aimed at promoter activity evaluation in suitable *in vivo* model systems, especially with pathogens such as *A. baumannii* and others. In *E. coli*, the ribose operon promoter was the strongest promoter we identified in this study. This was somewhat surprising because expression from this promoter is normally repressed by the ribose operon repressor RbsR and induced in the presence of the inducer D-ribose [47]. In this promoter-capture proof-of-concept study, we only examined *P*_*rbs*_-*lux* gene expression in LB-grown cells which must represent at least partially inducing conditions but the utility of this promoter for *in vivo* imaging of *E. coli* infections remains uncertain absent of expression studies in bacteria grown *in vivo*, e.g. animal infection or cell culture experiments, or *in vitro* studies employing various conditions encountered by bacteria during infections (e.g. cell-density, defined nutrient sources, etc.). The same is true for the *A. baumannii P*_*H1A*_ promoter identified and characterized using *in vitro* laboratory conditions, i.e. LB-grown bacteria. In addition to *E. coli* and *A. baumannii*, mini-Tn*5/7*-*lux* vectors were also used to identify strong promoters capable of driving *lux* operon expression in LB-grown cells of *B. pseudomallei*. Luminescent isolates could be readily identified suggesting that the system will be useful for promoter identification in diverse bacteria. Two promoters that were identified in *B. pseudomallei* were the *put* (proline utilization) and *paa* (phenylacetic acid degradation) operon promoters but since promoters for construction of bioluminescent *B. pseudomallei* suitable for *in vitro* and *in vivo* bioimaging studies, e.g. *P*_*ompA*_ [28] and *P*_*tolC*_ [25], are already available, the *put* and *paa* operon promoters were not further pursued.

Promoter identification in *E. coli*, *A. baumannii* and *B. pseudomallei* using the mini-Tn*5/7*-*lux* system identified a frequent scenario encountered with bacteria which is insertion in promoter-distal genes in operons. In practice, this makes direct use of mini-Tn*7*-*lux* elements with captured promoter regions for isolation of bioluminescent bacteria problematic in *recA*^*+*^ strain backgrounds as the sometimes large regions of homology carried by the transposable element promote recombination into the chromosome instead of site-specific integration via Tn*7*-transposition. While this undoubtedly diminishes the novelty of the mini-Tn*5/7*-*lux* system, i.e. the combination of the mini-Tn*5*-*lux* and mini-Tn*7*-*lux* systems which existed separately before, the newly developed method has several advantages over the separate systems: 1) the newly constructed pTn*7*Lux vectors exhibit expanded repertoire and utility with respect to cloning of promoter-containing DNA fragments when compared to previously constructed mini-Tn*7*-*lux* vectors; and 2) in some instances, e.g. where short promoter-containing chromosomal DNA regions are present or recombination-deficient strains are either available or can be readily constructed, the combination of the Tn*5* and Tn*7* transposon allows quick isolation and site-specific insertion of the promoter-*lux* fusion constructs in naturally occurring Tn*7* attachment site(s) in strains transiently expressing the Tn*7* TnsABCD transposase complex.

In the course of the present study we also noted that in the few instances where bacteria contain more than one chromosomal *att*Tn*7* site one must be aware of copy number and position effects on reporter gene expression. For instance, incorporation of the same mini-Tn*7*-*P*_*ompA*_-*lux* reporter into one or more of the three *att*Tn*7* sites in the *B. pseudomallei* genome resulted in differential levels of light emission. In general, insertions into the *glmS*1-associated *att*Tn*7* site emitted more light than mini-Tn*7* insertions in either of the other two *att*Tn*7* sites. Although we have no experimental evidence that would explain these observations, insertion site effects may at least be partially responsible for differential *lux* transcription from constructs integrated at different Tn*7* integration sites. The three *att*Tn*7* sites found in *B. pseudomallei* are located in the intergenic regions of *glmS1*, *glmS2* and *glmS3* and the respective downstream genes which in all cases are divergently transcribed from *glmS* (Additional file 1: Figure S3) [43]. The mini-Tn*7*-*lux* elements insert at these sites such that the *lux* gene is in the same orientation as these downstream genes which may lead to partial read-through *lux* transcription from the downstream gene promoters. This may be exacerbated by the fact that insertions at *glmS1* occur in the predicted transcriptional terminator that seems to be shared by *glmS1* and the divergently transcribed downstream gene. In contrast, insertions at the *glmS2*- and *glmS3*-associated *att*Tn*7* sites do not disrupt the transcriptional terminators of the respective divergently transcribed genes. To minimize transcriptional read-through effects from promoters of adjacent genes, transcriptional terminators could be included inside the Tn*7* left and right ends, but this was not pursued in the present studies. As expected, isolates with double insertions produced more light than those with single insertions and levels were comparable with isolates that contained single insertions at the *glmS1*-associated *att*Tn*7* site. Isolates with mini-Tn*7* insertions at all three *att*Tn*7* sites resulting from a single transposition experiment are generally rare and were not observed in this study.

## Conclusions

We created a suite of vectors that comprise a versatile system for promoter identification, capturing, cloning and construction of bioluminescent Gram-negative bacterial strains that contain the reporter genes stably integrated in the bacterial chromosome. The mini-Tn*5/7*-*lux* vectors incorporate the random transposition property of Tn*5* catalyzed by transient expression of Tn*5* transposase TnpA in a wide range of bacteria and combines it with the site-specific transposition property of Tn*7* catalyzed by transient expression of the Tn*7* TnsABCD transposase complex in Gram-negative bacteria. The system was created with versatility and customization in mind. For example, the vectors are equipped with diverse selection markers to expand their host range to bacteria, which may exhibit intrinsic resistance to some antibiotics commonly used for selection of recombinants. Antibiotic resistance markers are flanked by 48-bp *FRT* sites which allow exchange of the resident antibiotic marker with other *FRT* cassettes using unique *Xba*I restriction sites in each *FRT*. All vectors possess unique *Stu*I and *Dra*III restriction sites that allow for the deletion of *tnpA* and its flanking mosaic ends for orientation-controlled insertion of promoter sequences for *lux* operon transcription. In this study we exclusively tested Tn*5/7*Lux and Tn*7*Lux vectors for purposes of promoter identification, capturing and cloning for construction of bioluminescent clones. However, their uses extend well beyond these applications. For instance, *attB1* and *attB2* sites bordering *luxCDABE* facilitate exchange of the resident *lux* operon for other reporter genes such as *gfp* via Gateway BP clonase recombination. Vectors on which *gfp*-transcription is driven from the same promoter(s) identified and used for *lux* gene expression can then be employed for construction of fluorescent instead of luminescent strains. Availability of isogenic bioluminescent and fluorescent strains of the same species has several applications. For instance, they can be employed in bioluminescence, fluorescence, and optical density based real-time assays can to determine the bacteriostatic or bacteriocidal effects of antibiotics [53]. Furthermore, such strains can be used to differentiate effects of antimicrobials on metabolism. Luciferase activity is dependent on availability of metabolites such as ATP, FMNH_2_ and a specific fatty acid substrate [21] and its activity thus adversely affected by inhibitors of metabolism whereas GFP activity is not prone to such inhibition. Lastly, strain labeling with luciferase or GFP reporters – or dual labeling with both – broadens the repertoire for imaging of various biological processes [5,54].

These capabilities allow for tailoring the plasmids to investigators’ needs. The tools developed in this study should prove to be useful as their customizability allows for an extremely wide array of uses in diverse Gram-negative bacteria.

## Methods

### Bacterial strains, media and growth conditions

Table 2 lists the bacterial strains used in this study. Bacteria were routinely grown in liquid or agar solidified Lennox Luria Bertani (LB) (MO BIO Laboratories, Carlsbad, CA). *E. coli* conjugation strains RHO3 and RHO5 were grown in LB medium supplemented with diaminopimelic acid (DAP; LL-, DD-, and meso-isomers) which was used at 400 μg/ml for agar plates and 200 μg/ml for liquid cultures. Lennox (5 g/L NaCl) LB cultures of *B. pseudomallei* Bp82 were supplemented with 80 μg/ml adenine. Media were supplemented with antibiotics at the following final concentrations. For *E. coli*, gentamicin (Gm), 10 μg/mL and 15 μg/ml for broth cultures and agar plates, respectively; kanamycin (Km), 35 μg/mL; tetracycline (Tc), 10 μg/ml; trimethoprim (Tp), 100 μg/mL. For *B. pseudomallei,* Km, 35 μg/ml for Bp82.27 and 500 μg/ml for Bp82. For *A. baumannii*, Km was used at a concentration of 35 μg/ml.

### DNA manipulation

Chromosomal DNA was isolated using the Puregene Core Kit A (Gentra Systems, Qiagen, Valencia, CA) and plasmid DNA was purified from bacterial cultures using the GeneJET Plasmid MiniPrep Kit (Fermentas, Glen Burnie, MD). Restriction enzymes were purchased from New England Biolabs (Ipswich, MA) and used according to the manufacturer’s recommendations. Ligation reactions were conducted using T4 DNA ligase from Invitrogen (Life Technologies, Carlsbad, CA) and the supplied T4 DNA ligase buffer. DNA sequencing was conducted using an ABI 3130xL Genetic Analyzer (Applied Biosystems, Carlsbad, CA) at the Colorado State University Proteomics and Metabolomics Facility.

### Plasmid construction

Plasmid construction details are provided in Additional file 1: Methods and Table S1. The main plasmids constructed in this study are listed in Table 1.

### Transformation and conjugation procedures

Plasmid transformation of *E. coli* was done either by using standard electroporation or chemical transformation procedures [55]. Bacterial conjugations were conducted as bi-parental matings with *E. coli* mobilizer strains RHO3 or RHO5 using previously described methods [51,56]. A modified mating procedure was used for conjugations with *A. baumannii*. Cultures of donor and recipient were grown overnight. Thirty μl of the donor culture was sub-cultured into 3 ml of LB broth and the culture was grown at 37°C with shaking to an OD_600_ of 0.6-0.7. Meanwhile, 3 ml of pre-warmed 20 mM NaNO_3_ was added to the overnight recipient culture which was then incubated at 42°C without shaking for at least three hours. Donor and recipient cultures were then harvested by centrifugation, washed twice with fresh LB, concentrated 5-fold, and 60 μl of donor and 10 μl of recipient culture were combined on a filter disk. The remainder of the procedure follows previously described protocols.

### Construction and identification of mini-Tn*7*-*lux* containing *B. pseudomallei* strains

The mini-Tn*7*-*lux* delivery vectors pTn*7*oLuxK3, pTn*7*tLuxK3, pTn*7*xLuxK3 containing a *luxCDABE* operon transcribed from *P*_*ompA*_, *P*_*tolC*_ and *P*_*PA4974*_, respectively, and a Km^r^ selection marker were transformed into the *E. coli* mobilizer strain RHO3. The mini-Tn*7*-*lux* elements were delivered into the Bp82.27 recipient strain using multi-parental conjugation with RHO3 containing the Tn*7* transposase helper plasmid pTNS3 [43]. Km^r^ transformants were selected and mini-Tn*7*-*lux* insertion sites determined by PCR using primer sets P479 & P1509, P479 & P1510, and P479 & P1511 to check for insertion at *glmS*1, *glmS*2, or *glmS*3, respectively [43]. Table 3 lists oligonucleotides used in this study. Alternatively, a newly developed multiplex PCR was used to detect mini-Tn*7* inserts in *B. pseudomallei* and using the primer set P479 & 1510 & 2595 & 2596 (see Additional file 1: Methods for details and Additional file 1: Figure S3 for a representative example of multiplex PCR results).

**Table 3:** **Oligonucleotides**

**Oligo name**	**Description**	**Sequence (5′** → **3′)** ^**a**^
478	Tn*7*R	CACAGCATAACTGGACTGATTTC
479	Tn*7*L	ATTAGCTTACGACGCTACACCC
536	oriT-UP	TCCGCTGCATAACCCTGCTTC
537	oriT-DN	CAGCCTCGCAGAGCAGGATTC
572	PstSUp2	GCTATACGTGTTTGCTGATCAAGATGC
1354	AB_glmSF	GGCGGTCAGTTGTATGTCTT
1509	BPGLMS1	GAGGAGTGGGCGTCGATCAAC
1510	BPGLMS2	ACACGACGCAAGAGCGGAATC
1511	BPGLMS3	CGGACAGGTTCGCGCCATGC
2372	attTn7-1	GATGCTGGTGGCGAAGCTGTC
2373	attTn7-2	GATGACGGTTTGTCACATGGAG
2385	LUXpro-UP	ATTGCACTAAATCATCACTTTC
2550	Stu-PrbsEco-for	TAA*AGGCCT*GCCAGACGCCTCCTTTCT
2551	Dra-PrbsEco-rev	TAA*CACCGCGTG*TTCTCCATCAGCGAAACGT
2584	H1-A StuI F	ATT*AGGCCT*TCTTGCGAATCTTCTTCAATCTC
2586	H1A DraIII R3	ATT*CACCGCCTG*ACAAACTGAGATCCAACTCATACCT
2595	BPGLMS1-New4	ACCTGATTGCGTTCGTCGTCC
2596	BPGLMS3-New	ATCACGCTGCTTTGGCTGG

^a^Italicized letters indicate restriction sites: *Dra*III (2551 and 2586) and *Stu*I (2550 and 2584).

### Luminescence imaging

Relative luminescence was imaged using a Bio-Rad Universal Hood II ChemiDocXRS using high sensitivity chemiluminescence settings and a 10–30 s exposure time. Quantification of light production was performed using an IVIS Spectrum (Xenogen, Alameda, CA). An open emission filter with no excitation was utilized to measure the signal.

### Tn*5*/*7*-*lux* promoter capture procedure

To recover random mini-Tn*5/7-lux* chromosomal insertions, the Tn*5*/*7-lux* vector containing the appropriate antibiotic resistance marker was first transformed into the *E. coli* donor strain RHO3. A bi-parental mating was then performed with the donor and desired recipient strain and antibiotic resistant exconjugants were selected. Exconjugants were either patched onto an LB agar plate with the respective antibiotic used for selection of exconjugants or inoculated into 96-well microtiter plates containing LB medium with 10% glycerol and antibiotic supplement. After overnight growth at 37°C, patches or wells were observed over a period of four days to identify bright and stable luminescent clones. Chromosomal DNA was isolated from selected mini-Tn*5/7*-*lux* containing colonies and 1 μg digested in separate reactions using restriction enzymes *Acc*65I, *Eco*RI or *Not*I. (Note: these enzymes were empirically chosen to work with most bacteria we study but others can be used as well.) The digestions were terminated by heat-inactivation at 68°C and cleaned either by using the Gen Elute Gel Extraction Kit (Sigma-Aldrich) or by heat-inactivating the restriction enzyme and drop dialyzing the DNA on a Millipore“V” series (VMWP) filter with a 0.05 μm pore size over distilled and deionized water for 20 min. Digested DNA (1 μg) was ligated overnight using T4 DNA Ligase and ligations were drop dialyzed for 20–25 min. The entire sample was removed from the filter and immediately used for transformation of the *pir-116*^+^*E. coli* strain MaH1. (Note: other *pir*^+^ strains are equally suited for transformation. We routinely employ this strain because it increases the copy number of plasmid with the R6K origin of replication and thus yields more plasmid DNA [53].) Transformants were grown overnight and plasmid DNA was isolated. The mini-Tn*5/7-lux* insertion site was then determined by sequencing using primer P2385.

The resulting plasmid now constitutes a mini-Tn*7* delivery plasmid (Figure 1) which was used for two purposes.

First, the plasmid was sometimes used to isolate chromosomal mini-Tn*7*-*lux* insertions in the host of interest. This was achieved by electroporation into the *pir-116*^+^*E. coli* mobilizer strain RHO5 (electroporation was chosen over transformation due to the unknown, but presumably quite large, size of the recovered mini-Tn*5/7*-*lux* containing plasmid). The mini-Tn*7*-*lux* plasmid was then introduced into the target bacterium chromosome by co-conjugation with the Tn*7* site-specific transposition pathway expressing pTNS3. Exconjugants were selected on LB plates with appropriate antibiotics and screened for light production. The presence of mini-Tn*7*-*lux* insertions was verified by PCR employing species specific primer pairs, e.g. P2372 & P2373 for *E. coli*, P478 & P1354 for *A. baumannii*, and P479 & P1509, P479 & P1510, and P479 & P1511 for *B. pseudomallei*. To distinguish Tn*7* insertions at *att*Tn*7* sites from homologous recombination events a PCR using primer pairs P536 & P537 was also performed to confirm the absence of the plasmid-borne *oriT* in the recipient chromosome. All confirmatory PCRs were done using DNA templates obtained via from boiling preparations. Briefly, individual colonies were transferred to 30 μl of sterile distilled and deionized water and the cell suspension was boiled for 10 min. The resulting lysates were then centrifuged for 30 s at 12,000 × g at room temperature in a microcentrifuge and the supernatants transferred to a clean microcentrifuge tube. Six μl of supernatant were used a template in 50 μl PCR mixes containing the respective primers and *Taq* DNA polymerase (New England Biolabs).

Second, the mini-Tn*7*-*lux* plasmid was used as source for promoter-containing DNA fragments. Putative promoter regions were first predicted based on the genomic context of the insertion and using the Berkeley Drosophila Genome Project Neural Network Promoter Prediction and prokaryotic settings (http://www.fruitfly.org/seq_tools/promoter.html). Putative promoters were mapped onto the genome and the most likely promoter region was chosen based on number of possible promoters in the area and how close they were to the Tn*5* insertion. In general, oligonucleotides were designed to PCR amplify the promoter region and add DraIII and StuI restriction sites to either end to control the direction of the promoter upon cloning into the desired Tn*5*/7-*lux* vector (in each case the cloned promoter region replaced the Tn*5* transposase gene *tnpA* and flanking MEs). Transformants were chosen based on degree of luminescence and the presence of the correct plasmids was confirmed by a DraIII + StuI restriction digest and/or PCR amplification of the promoter region from the plasmid, followed by DNA sequencing. Mini-Tn*7*-*lux* insertions were then obtained and confirmed as described above.

## Additional file

Additional file 1:
**Additional methods; Table S1 Auxiliary plasmids used for vector construction; Figure S1 Genealogy of mini-Tn** ***7-*** ***lux*** **and mini-Tn** ***5/7-*** ***lux*** **vector creation from pTn** ***7*** **xLuxG0; Figure S2 Maps of** **mini-Tn** ***7*** **delivery vectors; Figure S3 Tn** ***7*** **insertion at multiple sites in** ***B. pseudomallei*** **and detection by multiplex PCR.**
