# Supplementary material for: Tn5/7-lux: a versatile tool for the identification and capture of promoters in Gram-negative bacteria
Source: BMC Microbiol. 2015 Feb 4;15(1):17. doi: 10.1186/s12866-015-0354-3 (PMC4328036; doi:10.1186/s12866-015-0354-3)
Supplement: Additional file 1: — Additional methods; Table S1 Auxiliary plasmids used for vector construction; Figure S1 Genealogy of mini-Tn 7- lux and mini-Tn 5/7- lux vector creation from pTn 7 xLuxG0; Figure S2 Maps of mini-Tn 7 delivery vectors; Figure S3 Tn 7 insertion at multiple sites in B. pseudomallei and detection by multiplex PCR. [file 12866_2015_354_MOESM1_ESM.pdf]

Additional methods

Linker tailing procedure. For linker tailing, approximately 1 µg of plasmid DNA was digested using the appropriate restriction enzyme(s) and cleaned using the Gen Elute Gel Extraction Kit (Sigma-Aldrich, St. Louis, MO). The non-phosphorylated linker oligonucleotides were pre-hybridized by mixing in sterile deionized water at a concentration of 0.1-0.5 µg/µl, boiling for 1 min, and cooling at room temperature for 2-3 h. Half of the digested plasmid DNA was ligated with 100X molar excess of linker. The ligations were conducted for either 4 h at room temperature or overnight at 14°C. The ligation mixtures were subsequently heated to 65-70°C for 5-10 min and then electrophoresed on a 1% agarose gel. The linear ligation product was purified from the gel using the Gen Elute Gel Extraction Kit (Sigma-Aldrich) and resuspended in 100 µl of hybridization buffer (10 mM Tris-HCl [pH 7.5], 100 mM NaCl, 1 mM EDTA). This mixture was then heated at 80°C for 5 min in a dry-block heater, the heater shut off and the solution slowly cooled overnight to room temperature. 10-20 µl of the annealing mixture was used directly for transformation.

Construction of pTn7xLuxG0. The mini-Tn*7* delivery vector pUC18-mini-Tn*7*T-GW- *lacPO-luxCDABE* is a plasmid for directional cloning of promoters to generate *Photorhabdus luminescens* luciferase transcriptional fusions [1]. It contains an *E. coli* lactose operon promoter (*lacPO*) fragment flanked by *Dra*III sites that directs lactose (or IPTG) inducible *luxCDABE* operon expression. The 3-nucleotide ambiguity in the *Dra*III recognition sites (5'-CAC**TAT**GTG and 5'- CAC**CGC**GTG) can be used for directional cloning of promoter-containing DNA fragments in place of the *lacPO* fragment. The *lacPO*-containing fragment of pUC18-mini-Tn*7*T-GW- *lacPO-luxCDABE* was replaced with the *P. aeruginosa* gene PA4974 promoter (P_{PA4974}) containing 475-bp DraIII fragment from pPS2305 (**Table S1**) to generate pUC18-mini-Tn*7*T-P_{PA4974}-*lux.* (The PA4974 promoter was identified in Xen5 which is a previously isolated *P. aeruginosa* strain which contains a mini-Tn*5*-*luxCDABE* insertion in the PA4974

gene whose promoter drives *lux* operon expression [2].) Next, a 806-bp cassette containing the R6K origin of replication (ori_{R6K}) and *oriT* was released from pPS2836 by digestion with *Sma*I and ligated with the *Bsr*BI digested backbone of pUC18-mini-Tn*7*T-P_{PA4974}-*lux* replacing the pUC origin of replication and the ampicillin resistance-encoding *bla* gene to create pTn*7*xLuxG0 (**Additional Fig. 1**). This plasmid contains the mini-Tn*7*T-P_{PA4974}-*lux* element with a gentamicin resistance selection marker (*aacC1*).

Construction of third generation plasmids. The construction of third generation plasmids originating from pTn*7*xLuxG0 is illustrated in **Additional Fig. 1**. First, we attempted to remove an extraneous *Xba*I site outside the multiple cloning site proximal *FRT* site of pTn*7*xLuxG0 by partial restriction digestion with *Bam*HI, followed by plasmid re-circularization and *Pst*I digest to remove background, creating pTn*7*xLuxG3. However, it was later determined that only the region between two *Pst*I sites residing in the multiple cloning site was removed. Thus, replacement of the gentamicin resistance marker of pTn*7*xLuxG3 with the *nptII*-encoded kanamycin resistance marker from pFKM4 [3] on an *Xba*I fragment resulted in the loss of one of the *FRT* sites flanking *nptII* in each resulting third generation vector (e.g. pTn*7*xLuxK3, pTn*7*oLuxK3, pTn*7*tLuxK3 and pTn*5/7*LuxK3).

The mini-Tn*5/7-lux* vector pTn*5/7*LuxK3 was created by *Dra*III+*Stu*I digestion of pTn*7*xLuxK3, followed by ligation with a *Dra*III-*Stu*I fragment containing the Tn*5* transposase encoding *tnpA* gene flanked by Tn*5* mosaic ends, replacing P_{PA4974}. The *tnpA* gene and ME-containing fragment was PCR amplified from pLG107 on a 1,978-bp fragment with primer pair 2427 & 2428. Clones containing *tnpA* were screened for by a significant loss of luminescence due to P_{PA4974} deletion and confirmed by a *Dra*III+*Stu*I restriction digest.

The P_{ompA} and P_{tolC} containing pTn*7*oLuxK3 and pTn*7*tLuxK3derivatives of pTn*7*oLuxK3 were derived by replacing P_{PA4974} with P_{ompA} and P_{tolC}, both of which were isolated on fragments obtained by PCR from

B. pseudomallei strain K96243 [4] chromosomal DNA using primer pairs 2378 & 2379 and 2380 & 2381, respectively. Directed P_{PA4974} replacement was achieved by incorporating *Dra*III sites with matching 3-nucleotide ambiguities to those on the vector on either side of the promoter-containing fragments.

To remove the unwanted *Xba*I site from pTn*7*xLuxG0, P_{ompA} was cloned into pTn7xLuxG0 to replace P_{PA4974} and its resident *Bam*HI site creating pTn*7*oLuxG0. The *Xba*I site was then removed by *Bam*HI digestion, followed by religation which created pTn*7*oLuxG4. Next, the gentamicin resistance marker was replaced by deleting the '*FRT-aacC1-FRT' Xba*I fragment with '*FRT-nptII-FRT'* (encoding neomycin phosphotransferase II) or '*FRT-dhfrII-FRT' Xba*I (encoding trimethoprim resistant dihydrofolate reductase II) fragments from pFKM2 [5] and pFTP2, respectively, creating pTn*7*oLuxK4 and pTn7oLuxT4. (The *'FRT* and *FRT'* indicate truncated *FRT* sites since *Xba*I cleaves within the *FRT* sequences.) The series of fourth generation of vectors was completed by constructing pTn*5/7*LuxG4, pTn5*/7*LuxK4 and pTn*5/7*LuxT4. This was achieved by replacing P_{ompA} with a 1,783-bp *Stu*I-*Dra*III fragment carrying the Tn*5* transposase *tnpA* gene flanked by the Tn5 mosaic ends. The *tnpA* gene is located upstream of the *lux* operon and oriented in the opposite orientation. Replacement of P_{ompA} was confirmed by loss of luminescence and *Dra*III+*Stu*I digests.

The construction of a fifth generation of vectors focused solely on the Tn*5*/*7*Lux plasmids. To increase *tnpA* transcription, the *B. thailandensis* ribosomal P_{S12} promoter was added to the *Eco*RI restriction site near the 5' end of the *tnpA* gene via linker tailing using oligonucleotides 2493 and 2494, creating pTn*5/7*LuxK5 and pTn*5/7*LuxT5 from pTn*5/7*LuxK4 and pTn*5/7*LuxT4, respectively. Insertion of P_{S12} was confirmed by sequencing. (The version containing a gentamicin marker was not pursued due to unexpected behavior of the vector, such as only being replicated in strains containing the *pir-116*$^+$ but not *pir*$^+$ allele.)

The sixth generation vector series originated from pTn*5/7*LuxT5. An *attB1* site was inserted into the *Dra*III site upstream of the *lux* operon via linker tailing with oligonucleotides 2540 and 2541 to allow for Gateway recombination for exchange of the *lux* operon with other genetic elements (an *attB2* site already existed downstream of *lux*). The insertion event creating pTn*5/7*LuxT6 was confirmed by sequencing. The remaining plasmids of this series – pTn*5/7*LuxK6, pTn*5/7*LuxG6 and pTn*5/7*LuxTc6 – were then derived from pTn*5/7*LuxT6 by replacing its *Xba*I-liberated '*FRT-dhfRII-FRT'* resistance cassette with '*FRT-nptII-FRT', 'FRT-aacC1-FRT'* and *'FRT-tetA-FRT'* cassettes from *Xba*I-digested pFKM2, pFGM1 and pFTC2, respectively.

Construction of empty mini-Tn*7* delivery vectors. Three empty mini-Tn*7* delivery vectors were constructed. To accomplish this, primer pair 2421 and 2422 was used to amplify the pTn7xLuxG3 backbone, excluding the *lux* operon, and add *Pst*I sites to either side of the amplicon. The amplified backbone was re-circularized to create pTn*7*xG (**Additional Fig. 2**). To replace the '*FRT-aacC1-FRT'* cassette, pTn7xG DNA was digested with *Xba*I and ligated with *Xba*I '*FRT-nptII-FRT'* and *'FRT-dhfrII-FRT'* fragments from pFKM4 and pFTP2 to derive pTn*7*xK and pTn*7*xT, respectively. These new mini-Tn*7* vectors contain unique restriction sites for cloning of cargo segments and the gentamicin, kanamycin and trimethoprim selection markers facilitate transposition selection in a broad-range of Gram-negative bacteria.

Multiplex PCR for detection of *B. pseudomallei* mini-Tn*7* *att*Tn*7* insertions. Because *B. pseudomallei* contains three *glmS*-associated *att*Tn*7* sites, the traditional method for determining the mini-Tn*7* insertion sites involved three separate PCR reactions employing a Tn*7*-specific primer and three chromosome-specific primers [5]. To streamline the insertion site detection method we developed a multiplex PCR using the primer set P479 & 1510 & 2595 & 2596. DNA templates used were obtained using colony boiling preparations. Briefly, individual colonies were transferred to 30 µl of sterile distilled

and deionized water and the cell suspension was boiled for 10 min. The resulting lysates were then centrifuged for 30 s at 12,000×g at room temperature in a microcentrifuge and the supernatants transferred to a clean microcentrifuge tube. Six μl of supernatant were used as template in 50 μl PCR mixes containing 30 pmoles of primers 479, 1510 and 2596 and 7.5 pmoles of primer 2595 in addition to *Taq* DNA polymerase (New England Biolabs). The PCR conditions employed were an initial denaturation step at 95°C for 2 min, 30 cycles of 95°C for 30 s, 61.2°C for 30 s and 72°C for 30 s, a final elongation step at 72°C for 10 min, followed by a hold at 8°C. Twenty μl aliquots of the PCR reaction mixtures were analyzed on a 1% agarose gel in TAE running buffer [6]. **Additional Fig. 3** is a representative example of multiplex PCR results.

Additional references

1. Moir DT, Di M, Opperman T, Schweizer HP, Bowlin TL: **A high-throughput, homogenous, bioluminescent assay for *Pseudomonas aeruginosa* gyrase inhibitors and other DNA damaging agents**. *J Biomol Scr* 2007, **12**:855-864.
2. Kadurugamuwa JL, Sin L, Albert E, Yu J, Francis K, DeBoer M, Rubin M, Bellinger-Kawahara C, Parr Jr TR, Jr., Contag PR: **Direct continuous method for monitoring biofilm infection in a mouse model**. *Infect Immun* 2003, **71**:882-890.
3. Kvitko BH, Bruckbauer S, Prucha J, McMillan I, Breland EJ, Lehman S, Mladinich K, Choi KH, Karkhoff-Schweizer R, Schweizer HP: **A simple method for construction of *pir*$^+$ Enterobacterial hosts for maintenance of R6K replicon plasmids**. *BMC Res Notes* 2012, **5**:157.
4. Holden MTG, Titball RW, Peacock SJ, Cerdeno-Tarraga AM, Atkins TP, Crossman LC, Pitt TL, Churcher C, Mungall KL, Bentley SD *et al*: **Genomic plasticity of the causative agent of melioidosis, *Burkholderia pseudomallei***. *Proc Natl Acad Sci USA* 2004, **101**:14240-14245.
5. Choi K-H, Mima T, Casart Y, Rholl D, Kumar A, Beacham IR, Schweizer HP: **Genetic tools for select agent compliant manipulation of *Burkholderia pseudomallei***. *Appl Env Microbiol* 2008, **74**:1064-1075.
6. Sambrook J, Russell DW: **Molecular Cloning**, Third edn. Cold Spring Harbor, NY: Cold Spring Harbor Laboratory Press; 2001.

7. Choi K-H, Gaynor JB, White KG, Lopez C, Bosio CM, Karkhoff-Schweizer RR, Schweizer HP: **A Tn*7*-based broad-range bacterial cloning and expression system.** *Nat Methods* 2005, **2**:443-448.

8. Waddell, CS, Craig NL: **Tn*7* transposition: Recognition of the *att*Tn*7* target sequence**. *Proc Natl Acad Sci USA* 1989, **86**:3958-3962.

Additional table 1. Auxiliary plasmids used for vector construction

Plasmid	GenBank Accession #	Pertinent features[a]	Source
pFGM1	AY597270	Ap^r, Gm^r; source of *FRT-aacC1-FRT* gentamicin resistance gene cassette	[7]
pFKM2	EU215433	Ap^r, Km^r; source of *FRT-nptII-FRT* kanamycin resistance gene cassette	[5]
pFKM4	JX504717	Ap^r, Km^r; source of *FRT-nptII-FRT* kanamycin resistance gene cassette	[3]
pFTP1	AY712951	Ap^r, Tp^r; source of *FRT-dhfRII-FRT* trimethoprim resistance gene cassette	[7]
pFTP2	KF532953	Ap^r, Tp^r; source of *FRT-dhfRII-FRT* trimethoprim resistance gene cassette; extra *Xba*I site within cassette removed by site-directed mutagenesis (TCTAGA → CCTAGA)	This study
pFTC1	AY712950	Ap^r, Tc^r; source of *FRT-tetA-FRT* tetracycline resistance gene cassette	[7]
pFTC2	KF532952	Ap^r, Tc^r; source of *FRT-tetA-FRT* tetracycline resistance gene cassette; extra *Xba*I site within cassette removed by site-directed mutagenesis (TCTAGA → CCTAGA)	This study
pLG107	N/A	Ap^r, Km^r; Tn*5* delivery vector; source of	Colin

		Tn*5* mosaic ends and *tnpA* encoded transposase	Manoil, University of Washington
pPS2305	KF532954	Ap^r; source of P_{PA4074}; pCR2.1 (Invitrogen) with 474-bp *Dra*III-P_{PA4974}-*Dra*III PCR fragment obtained with *P. aeruginosa* PAO1 chromosomal DNA and primers 1630 & 1631	This Study
pPS2836	KF532955	Ap^r; source of ori_{R6K}-*oriT* cassette; pCR2.1 with 817-bp PCR fragment obtained with pUC18R6KT-mini-Tn*7*T DNA and primers 2298 & 2299	This study
pTn*7*xLuxG0	KF532964	Gm^r; *luxCDEBA* operon transcribed from P_{PA4974} contained on *Dra*III fragment	This Study
pTn*7*oLuxG0	KF532961	Gm^r; *Dra*III fragment containing P_{PA4974} replaced with *Dra*III fragment containing P_{ompA}	This Study
pTn*7*xG	KF813058	Gm^r; pTn*7*xLuxG3 (Genbank accession no. KF532965) backbone amplified without *luxCDABE* operon; mini-Tn*7* delivery vector	This Study
pTn*7*xK	KF813059	Km^r; pTn*7*xG with Gm^r encoding *aacC1* gene replaced with Km^r encoding *nptII* gene from pFKM4	This Study

pTn*7*xT	KF813060	Tp^R; pTn*7*xG with Gm^r encoding *aacC1* gene replaced with Tp^r encoding *dhfRII* gene from pFTP2	This Study
pUC18R6KT-mini-Tn*7*T	AY712953	Ap^r; mini-Tn*7*T delivery vector	[7]
pUC18R6KT-mini-Tn*7*T-Km	KF532956	Ap^r Km^r; the released Km^r (*FRT-nptII-FRT*) encoding fragment from *Eco*53KI- digested pFKM2 was ligated with *Eco*RV-digested pUC18R6KT-mini-Tn*7*T.	This Study

[a]Abbreviations: Ap, ampicillin; Gm, gentamicin; Km, kanamycin; Tc, tetracycline; Tp, trimethoprim; *FRT*, Flp recombinase target.

Additional table 2. Oligonucleotides used in vector construction and Tn*7* insertion determination

Oligo Name	Description	Sequence (5' → 3')[a]
479	Tn*7*L	ATTAGCTTACGACGCTACACCC
1510	BPGLMS2	ACACGACGCAAGAGCGGAATC
1630	Xen5-Up	CACTATGTGATCGATGGCTCTCCTGGGTG
1631	Xen5-DW	CACCGCGTGTCGTTCGTCCTGGAAAAGATGC
2298	SmaI-R6KoriT-F	ATTCCCGGGAGGCCACCACTTCAAGAACTC
2299	Sma-R6KoriT-R	TAATCCCGGGCTTCCGCTTCCTCGCTCA
2378	DraIIIompA-UP	TAACACTATGTGAGACCGATGTTAGGGTGGGG
2379	DraIIIompA-DN	TAACACCGCGTGTGAGATTACCGCAGGTTTACTG
2380	DraIIItolC-UP	TAACACTATGTGGCGATTACAAGCCGGAATCAGGCT<br>ATCATGCACTCAAGTTG
2381	DraIIItolC-DN	TAACACCGCGTGGGTGAATTCAGGATCGTCAAAAAC<br>CGATATAAGACGGGACCG
2421	Tn7xFGmPstI-UP	ATAACTGCAGCACCGCGTGTCGTTCGTCCTG
2422	Tn7xFGmPstI-DN	ATAACTGCAGCTTTGCCTAATTGTAAGTGGA
2427	Stu-ME-TnpA-for	TAAAGGCCT**AGATGTGTATAAGAGACAG**ACTCTTCC<br>TTTTTCAATATTATTG
2428	DraIII-ME-tnpA-rev	TAACACCGCGTG**AGATGTGTATAAGAGACAG**GAATTC
2493	S12-Fwd	AATTCTTGACTCACTTGGGATTTTCGGAATATCATG
2494	S12-Rev	AATTCATGATATTCCGAAAATCCCAAGTGAGTCAAG
2540	DraIIIattB1 F	GTGACAAGTTTGTACAAAAAAGCAGGCTGCG

2541	DraIIIattB1R	AGCCTGCTTTTTTGTACAAACTTGTCACCGC
2595	BPGLMS1-New4	ACCTGATTGCGTTCGTCGTCC
2596	BPGLMS3-New	ATCACGCTGCTTTGGCTGG

[a]Restriction sites are underlined: *Dra*III (1630, 1631, 2738, 2739, 2380, 2381, 2428); *Pst*I (2421 and 2422); *Sma*I (2298, 2299); *Stu*I (2427). The Tn*5* mosaic end sequences in oligonucleotides 2427 and 2428 are bolded.

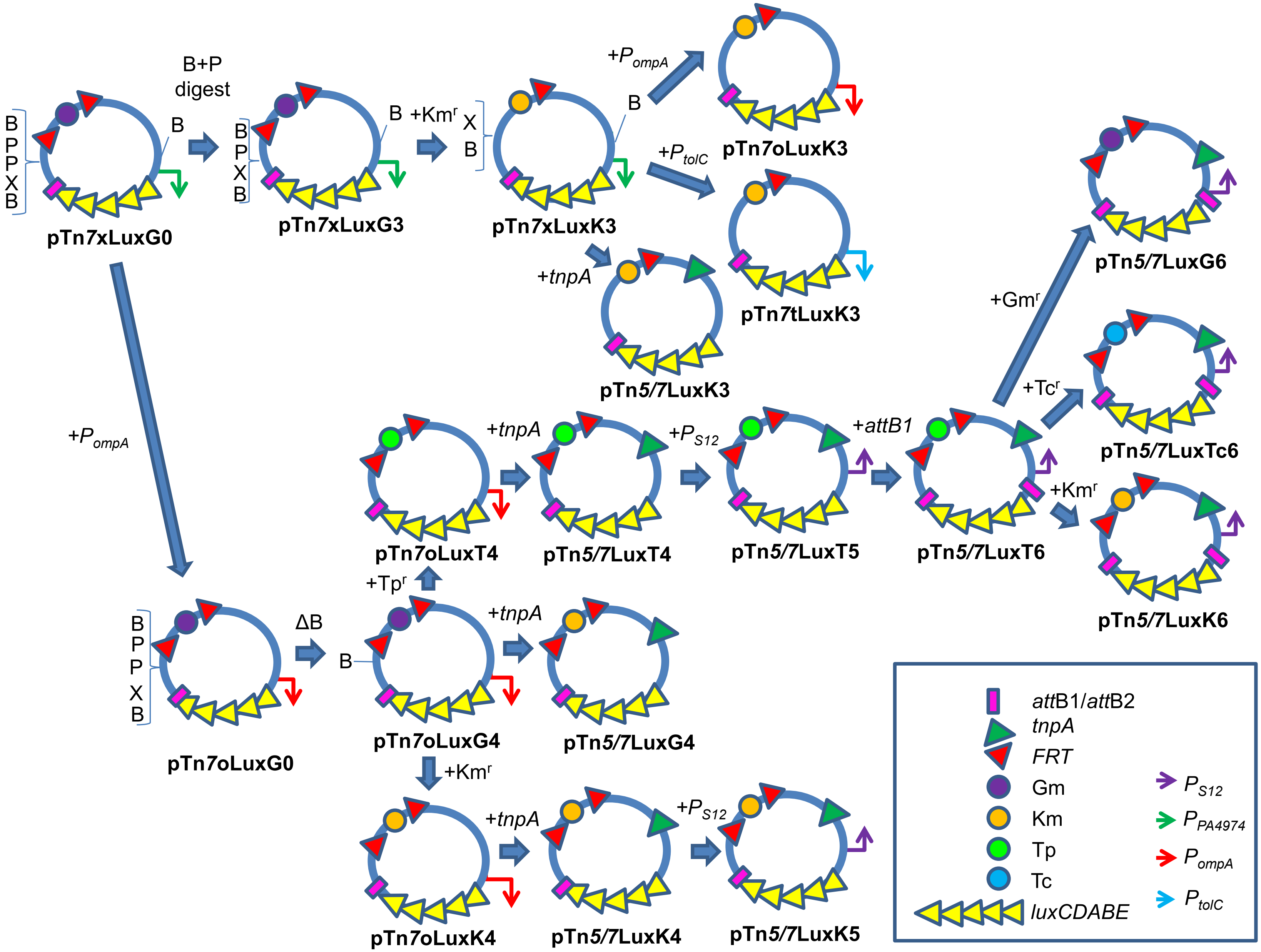

B+P digest
B
P
P
X
B
pTn7xLuxG0
pTn7xLuxG3
+Km^r
pTn7xLuxK3
+P_{ompA}
pTn7oLuxK3
+P_{tolC}
pTn7tLuxK3
+tnpA
pTn5/7LuxK3
pTn5/7LuxG6
+Gm^r
+Tc^r
pTn5/7LuxTc6
+Km^r
pTn5/7LuxK6
+attB1
pTn5/7LuxT6
+P_{S12}
pTn5/7LuxT5
pTn5/7LuxT4
pTn7oLuxT4
+Tp^r
pTn7oLuxG0
ΔB
pTn7oLuxG4
pTn5/7LuxG4
pTn7oLuxK4
pTn5/7LuxK4
pTn5/7LuxK5
attB1/attB2
tnpA
FRT
Gm
Km
Tp
Tc
luxCDABE
P_{S12}
P_{PA4974}
P_{ompA}
P_{tolC}

Additional figure 1. Genealogy of mini-Tn*7-lux* and mini-Tn*5/7-lux* vector creation from pTn*7*xLuxG0. Plasmid nomenclature indicates what the respective vectors carry and the generation they belong to. For example capital letters G, K, T and Tc indicate gentamicin, kanamycin, trimethoprim and tetracycline resistance markers used for either mini-Tn*5/7-lux* or mini-Tn*7-lux* transposition selection. Letters 3 to 6 indicate vector generation and lower case letters o, t and x indicate the *B. pseudomallei ompA* (P_{ompA}), *B. pseudomallei tolC* (P_{tolC}) and *P. aeruginosa PA4974* (P_{PA4974}) promoters, respectively. Letters B, P and X indicate *Bam*HI, *Pst*I and *Xba*I restriction sites. B+P denotes a *Bam*HI partial and *Pst*I complete restriction digest and ΔB indicates a *Bam*HI deletion. Steps marked with +Gm^r, +Km^r, +Tp^r and +Tc^r indicate replacement of the resident resistance marker with gentamicin (*aacC1*), kanamycin (*nptII*), trimethoprim (*dhfRII*) and tetracycline (*tetA*) resistance markers. Steps marked with +*tnpA* indicate addition of the Tn*5* transposase gene with flanking Tn*5* mosaic ends and steps marked with +*attB1* mark addition of a bacteriophage λ *attB1* recombination site. Steps marked with +P_{S12} indicate insertion of a *B. thailandensis* ribosomal S12 gene promoter. Transcription directions from promoters are indicated with an arrow. Color coding of genetic elements is explained in the insert box.

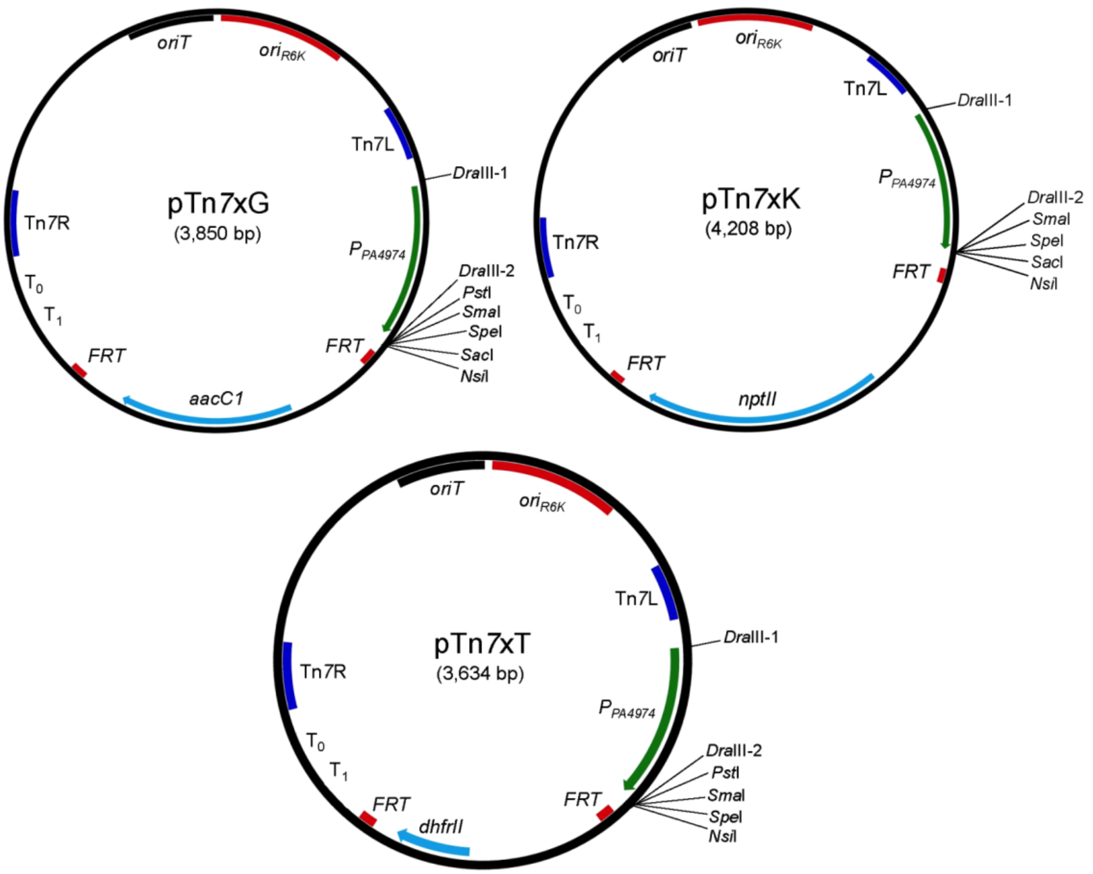

Additional figure 2. Maps of mini-Tn*7* delivery vectors. The mini-Tn*7* elements on these delivery vectors are flanked by the Tn*7* left (Tn*7*L) and right (Tn*7*R) ends and contain a either an *aacC1*-encoded gentamicin resistance marker (pTn*7*xG), a *nptII*-encoded kanamycin resistance marker (pTn*7*xK) or a *dhfRII*-encoded trimethoprim resistance marker (pTn*7*xT). The resistance genes are flanked by Flp recombinase targets (*FRT*). The mini-Tn*7* element also contains the transcriptional terminators T_0 and T_1 from bacteriophage λ and *E. coli rrnB* operon, respectively, and the promoter from the *P. aeruginosa* PA4974 gene (P_{PA4974}). The plasmid backbone contains the R6K origin of replication (*ori*$_{\mathrm{R6K}}$) and an origin of conjugative transfer (*oriT*). Unique restriction sites for cloning of cargo fragments are shown. The two *Dra*III sites contain a 3-nucleotide ambiguity in the recognition sites (*Dra*III-1 5'-CAC**TAT**GTG and *Dra*III-2 5'- CAC**CGC**GTG) and aside from deletion of P_{PA4974} can therefore also be used for directional cloning of promoter-containing DNA fragments for transcription of cargo genes.

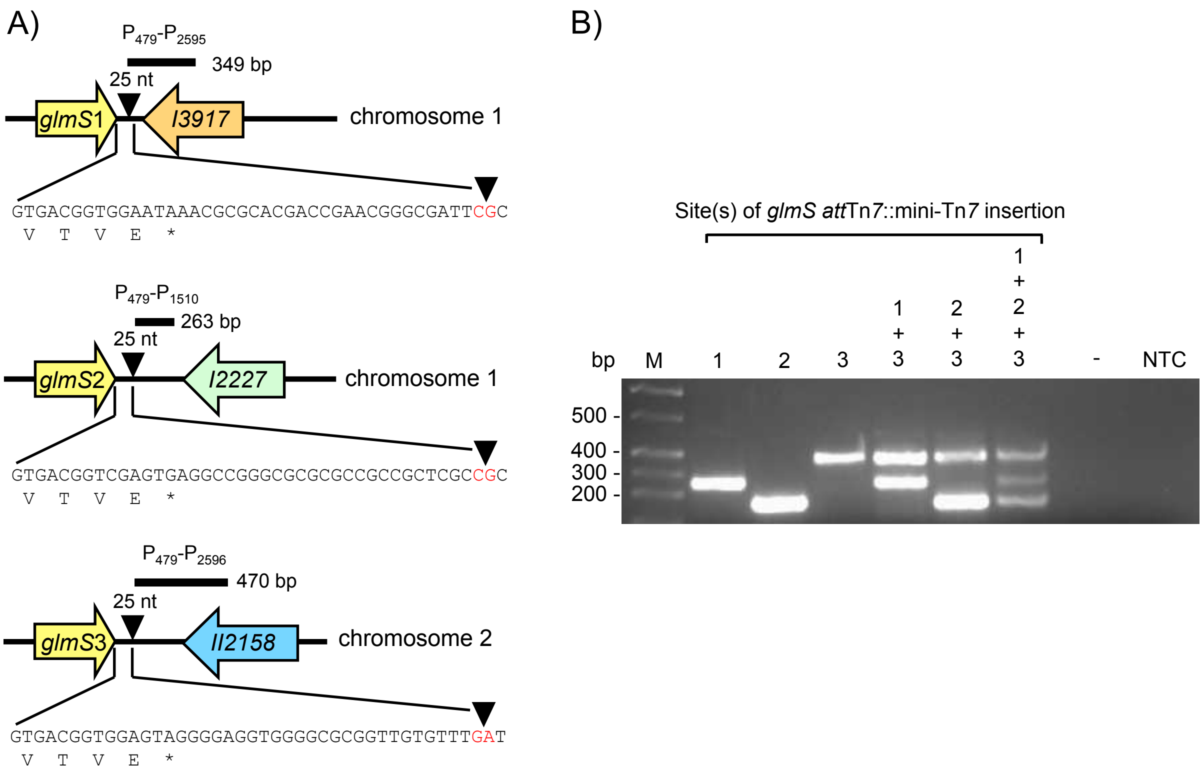

Additional figure 3. Tn*7* insertion at multiple sites in *B. pseudomallei* and detection by multiplex PCR. (**A**) Insertion occurs at three possible *glmS*-linked *att*Tn*7* sites, two on chromosome 1 and one on chromosome 2, but >65% of observed insertions occur at the *glmS2*-associated site on chromosome 1. Although the *att*Tn*7* target sequences are located within the last 40 nucleotides of the *glmS* coding sequences Tn*7* elements insert 25 nucleotides (25 nt) downstream of the respective *glmS* gene in the *glmS*-downstream gene intergenic region [8]. The downstream genes are labeled with the short form of their respective strain Bp82 annotations as BP1026B_I3917, BP1026B_I2227 and BP1026B_II2158. The sequences shown include the last five codons of the respective *glmS* genes and the Tn*7* insertion sites marked by triangles and nucleotides in red type. Expected PCR fragment sizes (taking into account the 5 bp insertion site duplication [8]) are indicated by horizontal bars, which are labeled with primer pairs used for amplification and fragment sizes. (**B**) Representative multiplex PCR results of verification of mini-Tn*7* insertion at one or multiple *B. pseudomallei* genomic insertion sites. Lanes labeled 1, 2 and 3 indicate mini-Tn*7* insertion at *glmS1*-, *glmS2*- or *glmS3*-associated *att*Tn*7* sites. Combinations of numbers indicate insertions at multiple *att*Tn*7* sites. For example, 1+2+3 indicates insertions at all three sites. **Other lanes:** – , DNA from Bp82.27 host strain without mini-Tn*7* insertion; NTC**,** no template control. The lane labeled M contained Hi-Lo molecular weight markers from Minnesota Molecular (Minneapolis, MN).
